# Supplementary material for: Auditory stimulation during REM sleep modulates REM electrophysiology and cognitive performance
Source: Commun Biol. 2024 Feb 16;7:193. doi: 10.1038/s42003-024-05825-2 (PMC10873307; doi:10.1038/s42003-024-05825-2)
Supplement: Supplementary file 2 — Supplementary Information [file 42003_2024_5825_MOESM2_ESM.pdf]

Supplemental Information

Auditory stimulation during REM sleep modulates REM electrophysiology and cognitive performance.

Miguel Navarrete, Viviana Greco, Martyna Rakowska, Michele Bellesi, Penelope A. Lewis

*Correspondence:

Miguel Navarrete: mnavarretem@gmail.com

Penelope A. Lewis: LewisP8@cardiff.ac.uk

Supplementary Figures

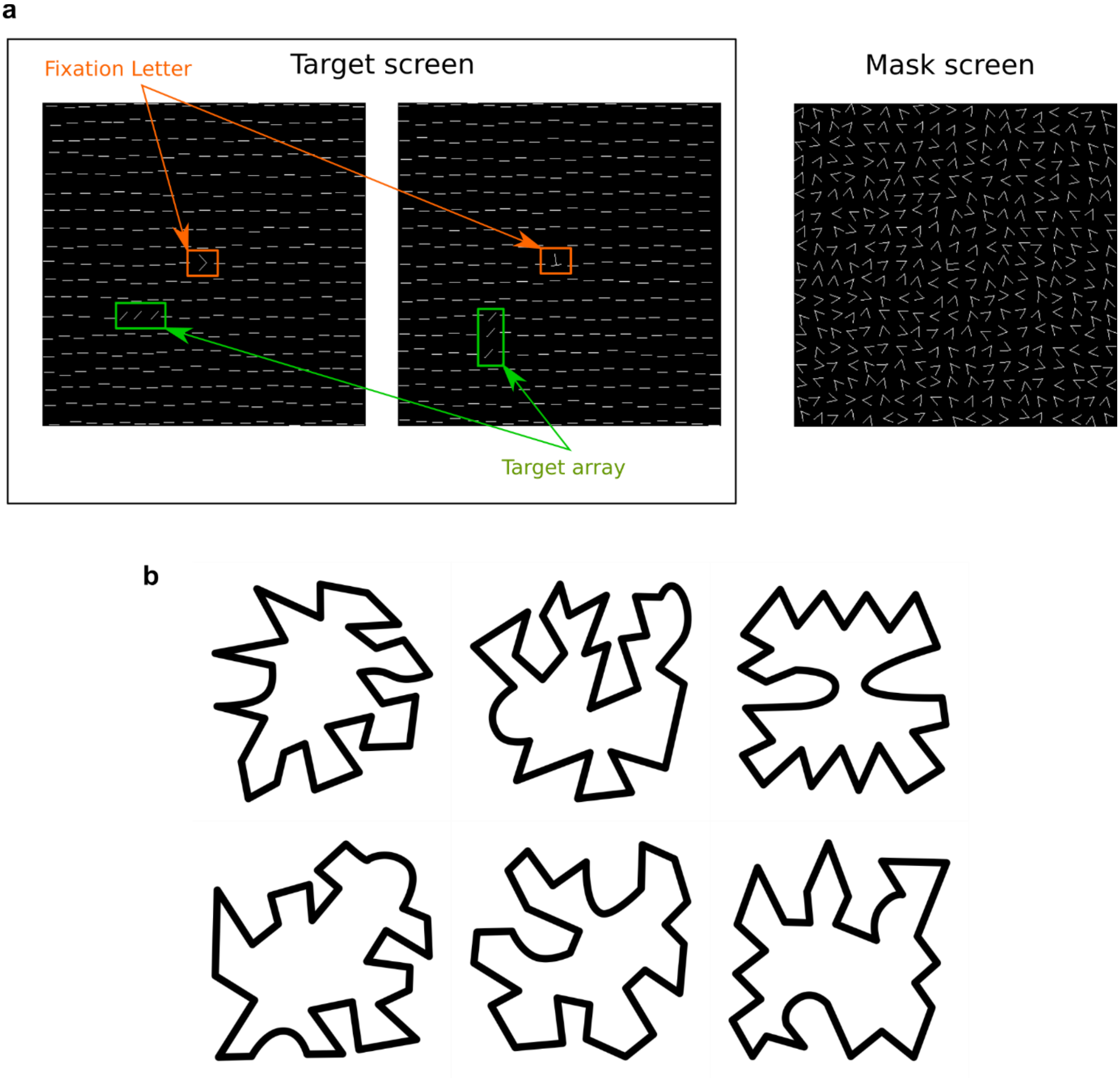

Supplementary Figure 1. Behavioural tasks. a) Visual texture discrimination test (VDT). LEFT: Two examples of target screens, containing two pieces of information: (1) a fixation letter, i.e., a rotated “T” or “L” at the centre of the screen; (2) a target array of three vertical or horizontal diagonal lines located in one of the four quadrants of the screen (here presented in the left-lower quadrant). RIGTH: a mask screen with scrambled lines was presented after the target screen. b) Examples of tracing shapes for the mirror tracing test (MTT). These consisted of complex closed figures made up of 26 to 27 angles enclosing straight segments and one or two curved sections. The figures were randomly assigned to each subject for test-retest in each night, but not repeated between nights.

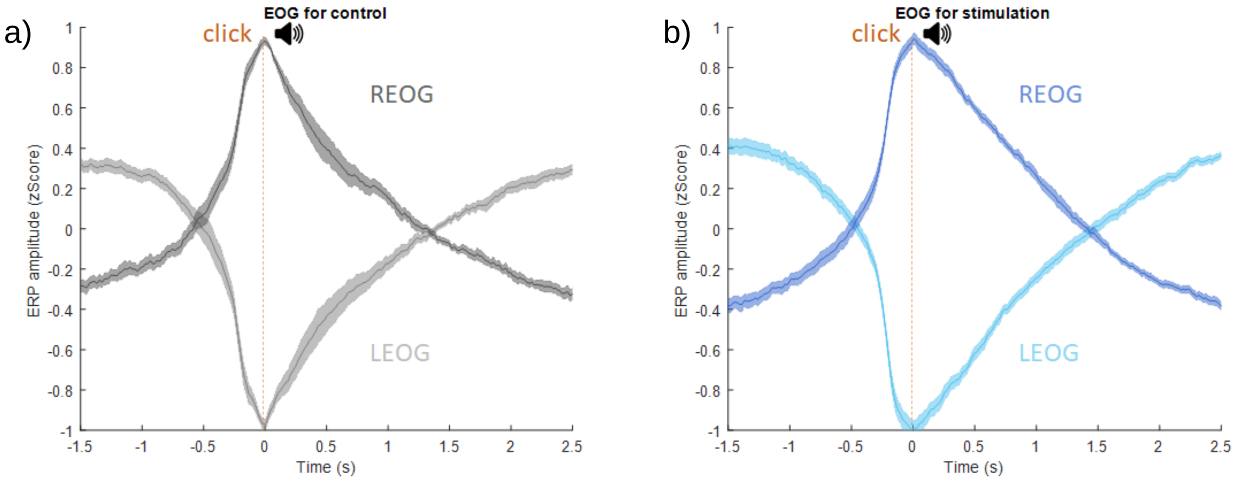

Supplementary Figure 2. Auditory clicks were locked to eye-movements. Average EOG signal for control (a) and stimulation (b) trials locked to the clicks suggests that the stimulation was indeed applied during the eye movements. REOG: right EOG; LEOG: left EOG. All error bars represent mean ± 95% CI.

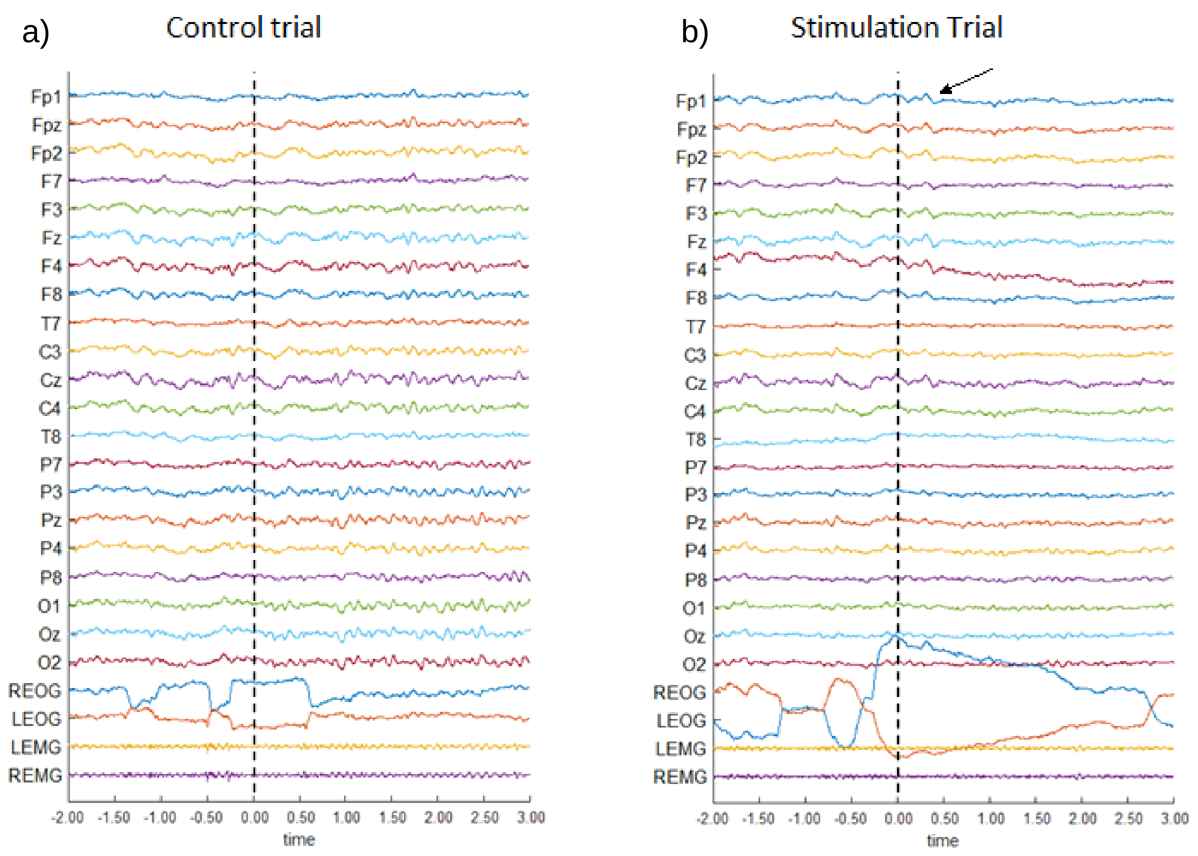

Supplementary Figure 3. Example of a control (a) and a stimulation (b) trial from the same participant. An ERP response is visible at the fronto-central channels upon stimulus presentation (arrow), followed by beta components.

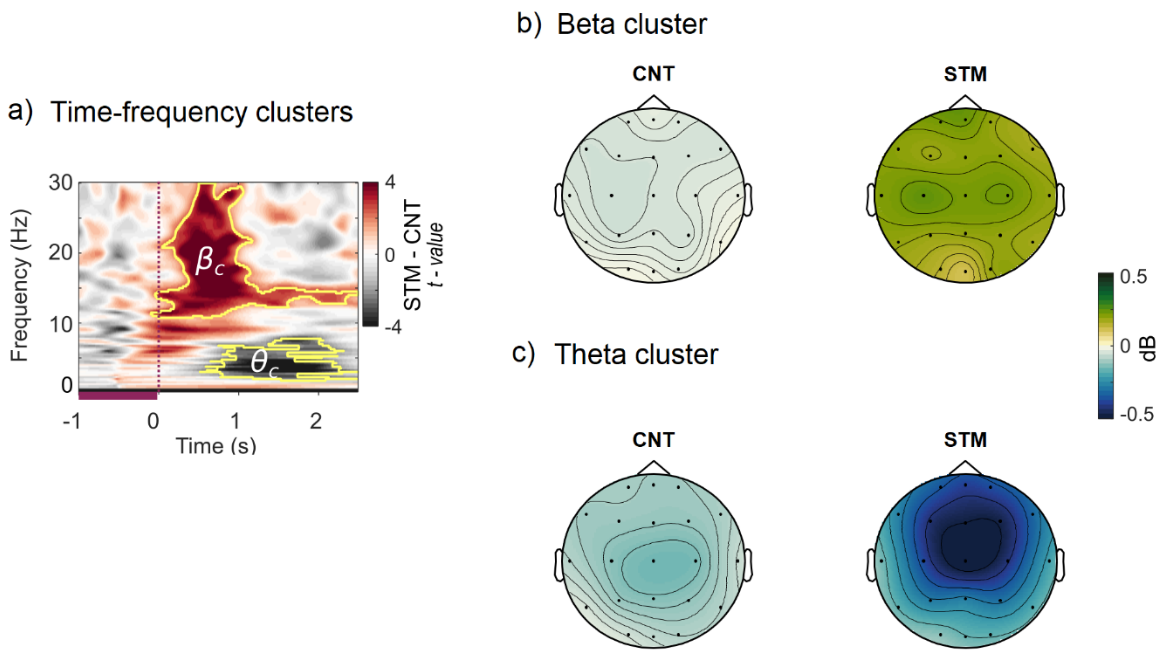

Supplementary Figure 4. Topographic distribution of beta and theta clusters. a) Analysis of spectral differences between conditions indicated two main clusters in beta (βc) and theta (θc) frequencies. A darker purple bar between at -1 and 0 seconds below the time-frequency plot indicates the baseline period. Topographic distribution of the beta (b) and theta (c) clusters in CNT (left) and STM conditions (right) expresed in dB from the baseline period.

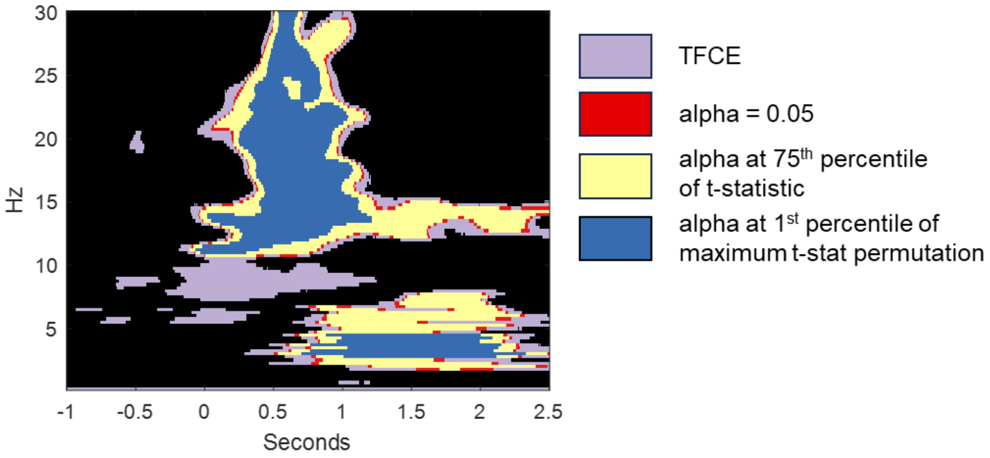

Supplementary Figure 5. Beta and theta clusters under different threshold selections. Spectral Beta and Theta clusters are evidenced when using different thresholding methods. From least to most strict, the figure shows overlapped significative clusters when using thresholdings from methods: Purple cluster, Threshold-free cluster enhancement (TFCE; E = 0.2, H = 2); Red cluster, alpha thresholding (used in our analyses); Yellow cluster, alpha at the 75th percentile of the t-statistic; blue cluster, alpha at 1st percentile of the pemutation of the maximum t-statistic.

.

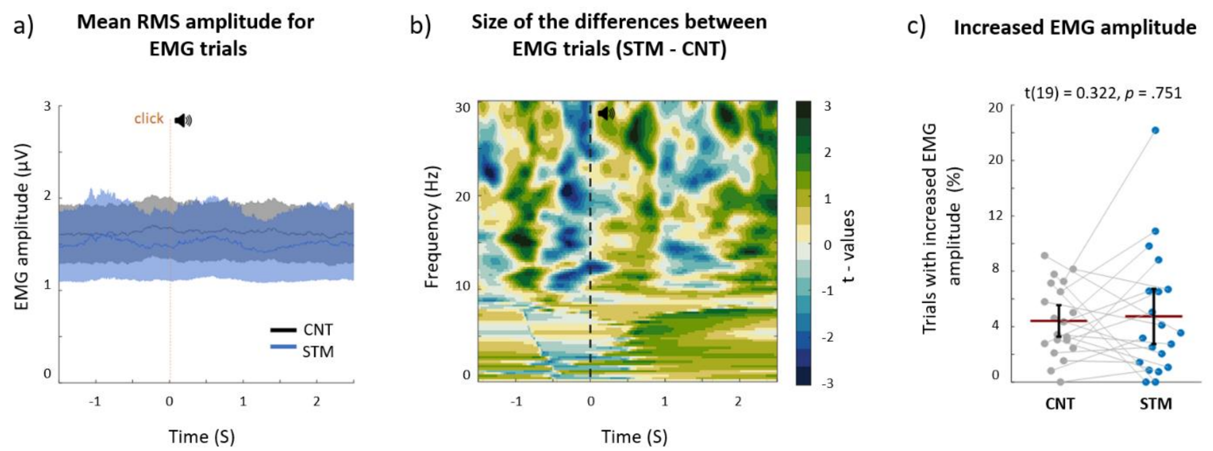

Supplementary Figure 6. Clicks locked to eye-movements do not increase REM arousals. a) Root mean squared (RMS) amplitude (filtered in the 10-100 Hz band) of the submental EMG in control (grey) and stimulation (blue) trials. **b)** No difference was revealed between the control and stimulation conditions in terms of the EMG spectral clusters following acoustic click. **c)** Percentage of trials for each subject/condition where an increase in EMG amplitude was observed upon stimulus presentation. An increase in EMG amplitude was defined as the RMS signal surpassing the mean + 3.5 SD of the RMS amplitude during REM. The increasing of submental EMG amplitude due to stimulation was computed as the percentage of trials for which the RMS of the EMG amplitude surpassed the threshold within 0 – 2 s following the sound click. We found no differences in the percentage of trials with increased EMG amplitude between the conditions of control (CNT) and stimulation (STM). All error bars represent mean ± 95% CI.

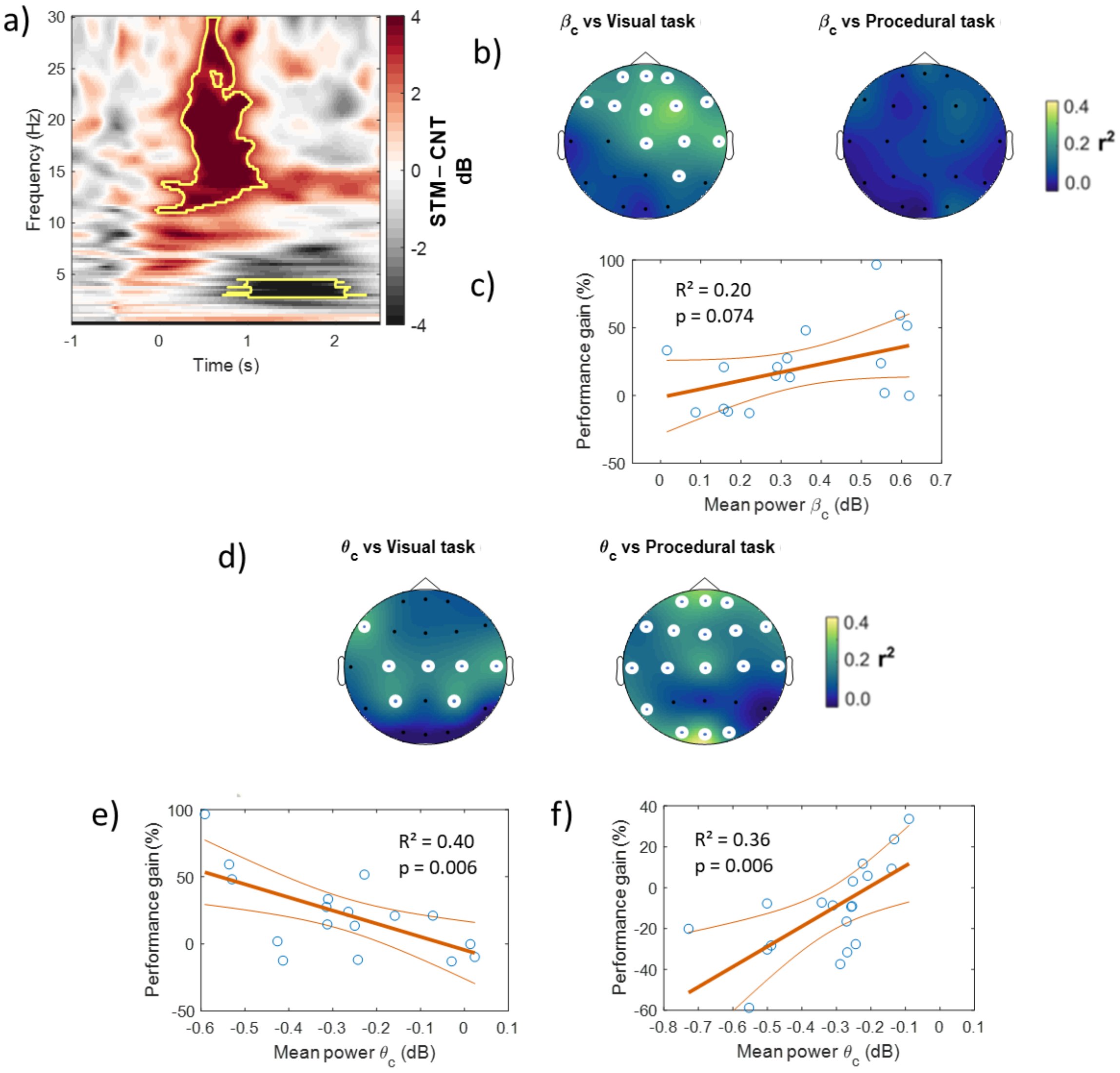

Supplementary Figure 7. **Spectral response to auditory stimuli of REM using stricter cluster thresholding**. a) Mean event-related spectral perturbations (ERSP) across all channels in response to the auditory click. As in the original analysis, a stricter thresholding (determined as the 1st percentile of the permutation of the maximum t-statistic) find spectral differences between conditions indicated two main clusters in beta (βc) and theta (θc) frequencies. Changes of power within βc (b) and θc (d) for each electrode fit to linear regressions predicting overnight improvement in the performance of the visual (VDT) and procedural (MTT) tasks. White channels indicate significant linear fits after FDR correction ($q < .05$). (c) Associations between βc and overnight performance gain for the visual task. (e) Associations between θc and overnight performance gain for the visual task. (f) Associations between θc and overnight performance gain for the performance task.

Supplementary Tables

Supplementary Table 1. Click stimulation accuracy for REM sleep and EM events.

	mean	std	median	IQR
**ACC for clicks during REM sleep.**	88.69	12.86	92.52	10.61
**ACC for clicks on EM events during sleep time ([a]).**	30.07	14.53	32.22	21.63
**ACC for clicks on EM events during REM.**	33.24	15.66	36.71	24.62

EM = Eye movements. ACC: Accuracy = 100 × (Click Events) / (Total events). ([a]) Including REM, NREM, and WASO.

Supplementary Table 2. Sleep characteristics and differences between experimental nights.

Variables	CNT	STM	*p*
Detected events (trials)	350.79 ± 239.39	182.95 ± 98.96	**.004**
Trials during REM sleep (%)	81.87% ± 8.80%	85.49 % ± 15.19%	.276
Accepted trials in phasic REM (%)	73.42% ± 8.15%	77.00 % ± 14.07%	.278
Total number of EM	981.05 ± 956.58	870.90 ± 810.79	.292
Stimulated EM during phasic REM (%)	38.82% ± 26.22%	26.69% ± 25.78%	.185
EM inter-event interval (s)	12.02s ± 4.44s	17.84s ± 10.47s	.057

Note: N = 19 subjects for each condition. EM = Eye movements during REM sleep. Values are given as mean ± standard deviation. All p-values are uncorrected.

Supplementary Table 3. Summary of regression analysis for sleep stages predicting performance gain on the visual task. Multivariable linear regressions were defined as %Change = $\beta_0 + \beta_1\,(Condition) + \beta_2\,(Sleep\ Stage)$.

		Coefficients		
**Variable**		***β***	***SE***	***p***
Model in N1				
Intercept		-10.10	6.46	0.1270
Condition (STM vs CNT)		17.71	5.99	**0.0056**
Stage N1 (min)		0.12	0.10	0.2627
R^2	0.22			
F for change in R^2	4.65			**0.0163**
Model in N2				
Intercept		-6.30	12.83	0.6265
Condition (STM vs CNT)		16.17	6.24	**0.0140**
Stage N2 (min)		0.01	0.07	0.8633
R^2	0.18			
F for change in R^2	3.66			**0.0362**
Model in N3				
Intercept		0.87	9.52	0.9279
Condition (STM vs CNT)		17.22	6.06	**0.0075**
Stage N3 (min)		-0.05	0.07	0.5348
R^2	0.19			
F for change in R^2	4.08			**0.0259**
Model in NREM (N2 & N3)				
Intercept		6.21	21.78	0.9655
Condition (STM vs CNT)		17.89	6.45	**0.0092**
Stage NREM (min)		-0.04	0.07	0.6341
R^2	0.19			
F for change in R^2	4.00			**0.0275**
Model in REM				
Intercept		0.49	11.18	0.9655
Condition (STM vs CNT)		16.90	6.12	**0.0092**
Stage REM (min)		-0.06	0.13	0.6341
R^2	0.19			
F for change in R^2	3.86			**0.0308**

Note: N = 18 subjects for Visual Task, Error degrees of freedom: 34. Significant p values in bold.

Supplementary Table 4. Summary of regression analysis for sub-stages of REM sleep predicting performance gain on the visual task. Multivariable linear regressions were defined as %Change = $\beta_0 + \beta_1$ *(Condition)* + β_2 *(Sleep Sub-stage).*

Variable		Coefficients β	*SE*	***p***
Model in tonic REM				
Intercept		13.46	8.57	0.1257
Condition (STM vs CNT)		20.50	6.39	**0.0029**
Tonic REM (min)		-0.66	0.25	**0.0140**
R^2	0.3			
F for change in R^2	7.17			**0.0025**
Model in phasic REM				
Intercept		-12.51	9.09	0.1779
Condition (STM vs CNT)		16.64	5.90	**0.0079**
Phasic REM (min)		0.17	0.16	0.3149
R^2	0.21			
F for change in R^2	4.46			**0.0191**
Model in % REM				
Intercept		-41.88	16.82	0.0969
Condition (STM vs CNT)		19.29	6.19	**0.0037**
Phasic REM (% of REM sleep)		0.60	0.26	**0.0264**
R^2	0.27			
F for change in R^2	6.36			**0.0045**

Note: N = 18 subjects for Visual Task, Error degrees of freedom: 34. Significant p values in bold.

Supplementary Table 5. Summary of regression analysis for sleep stages predicting performance gain on the procedural task. Multivariable linear regressions were defined as %Change = $\beta_0 + \beta_1$ *(Condition)* + β_2 *(Sleep Stage)*.

**Variable**		Coefficients β	*SE*	*p*
Model in N1				
Intercept		20.53	5.40	**0.0005**
Condition (STM vs CNT)		-12.97	5.08	**0.0151**
Stage N1 (min)		-0.05	0.09	0.6028
R^2	0.16			
F for change in R^2	3.47			**0.0418**
Model in N2				
Intercept		23.42	11.18	**0.0433**
Condition (STM vs CNT)		-12.38	5.29	**0.0249**
Stage N2 (min)		-0.03	0.06	0.6440
R^2	0.16			
F for change in R^2	3.34			**0.0467**
Model in N3				
Intercept		14.64	8.34	0.0875
Condition (STM vs CNT)		-12.58	5.27	**0.0224**
Stage N3 (min)		0.03	0.06	0.6416
R^2	0.14			
F for change in R^2	3.01			0.0616
Model in NREM (N2 & N3)				
Intercept		17.09	18.88	0.3715
Condition (STM vs CNT)		-12.60	5.51	**0.0282**
Stage NREM (min)		0.00	0.06	0.9530
R^2	0.14			
F for change in R^2	2.96			0.0646
Model in REM				
Intercept		33.01	9.13	**0.0009**
Condition (STM vs CNT)		-11.43	4.96	**0.0270**
Stage REM (min)		-0.19	0.11	0.0830
R^2	0.21			
F for change in R^2	4.74			**0.0149**

Note: N = 20 subjects for Procedural Task, Error degrees of freedom: 36. Significant p values in bold.

Supplementary Table 6. Summary of regression analysis for sub-stages of REM sleep predicting performance gain on the procedural task. Multivariable linear regressions were defined as %Change = $\beta_0 + \beta_1$ *(Condition)* + β_2 *(Sleep Sub-stage).*

**Variable**		**Coefficients** β	***SE***	***p***
Model in tonic REM				
Intercept		19.01	7.34	**0.0138**
Condition (STM vs CNT)		-12.44	5.32	**0.0250**
Tonic REM (min)		-0.03	0.21	0.8960
R^2	0.14			
F for change in R^2	3.00			0.0625
Model in phasic REM				
Intercept		31.90	7.35	**0.0001**
Condition (STM vs CNT)		-12.04	4.79	**0.0165**
Phasic REM (min)		-0.28	0.13	**0.0364**
R^2	0.24			
F for change in R^2	5.60			**0.0076**
Model in % REM				
Intercept		35.79	14.51	**0.0185**
Condition (STM vs CNT)		-12.83	5.29	**0.0204**
Phasic REM (% of REM sleep)		-0.29	0.22	0.1959
R^2	0.16			
F for change in R^2	3.42			**0.0437**

Note: N = 20 subjects for Procedural Task, Error degrees of freedom: 36. Significant p values in bold.

Supplementary Table 7. Correlations between behavioural tasks and click accuracy (%).

	% EMs targeted during *REM sleep*	% Clicks on EMs during *total sleep time*	% Clicks on EMs during *REM sleep time*
VDT change	-0.27 (0.282)	0.00 (0.999)	0.10 (0.695)
MTT change	-0.43 (0.067)	**-0.57** (0.011)	**-0.54** (0.016)
PVT change	0.25 (0.311)	-0.09 (0.720)	-0.13 (0.594)

Note: N = 18 subjects for Visual Task, N = 20 subjects for Procedural tasks. Values indicate Pearson's ρ correlations and associated p values in parenthesis. Bold values indicate significant correlations at $p < 0.05$.

Supplementary Table 8. Correlations between behavioural tasks and PANAS scores.

	Visual Task		Procedural Task		Attention Task	
	CNT	STM	CNT	STM	CNT	STM
Positive Affect Score	0.28	0.21	0.18	-0.01	0.37	-0.01
Negative Affect score	0.35	-0.18	-0.17	-0.07	0.14	0.24

Note: N = 18 subjects for Visual Task, N = 20 subjects for Procedural and Attention tasks. Values indicate Pearson's ρ correlations. All $p > 0.05$.

Supplementary Table 9. Correlations between behavioural tasks and relative power clusters

	Visual Task		Procedural Task	
	CNT	STM	CNT	STM
Relative β_c power	0.10 (.701)	**0.54** (.017)	0.15 (.541)	-0.09 (.697)
Relative θ_c power	-0.28 (.265)	-0.38 (.108)	**0.57** (.011)	0.11 (.640)

Note: N = 18 subjects for Visual Task, N = 20 subjects for Procedural tasks. Values indicate Pearson's ρ correlations and associated p values in parenthesis. Bold values indicate significant correlations at $p < 0.05$.

Supplementary Table 10. Uncorrected and corrected P-values for linear regressions associating Beta Power vs Change of task performance for each electrode.

Uncorrected p values									
Beta - Visual Task					*Beta - Procedural Task*				
	0.0046	0.0034	0.0020			0.1303	0.0279	0.1036	
0.0035	0.0080	0.0014	0.0015	0.0153	0.0293	0.2787	0.1403	0.0965	0.1861
0.1146	0.0196	0.0010	0.0012	0.0012	0.1611	0.1115	0.0953	0.0323	0.1951
0.0703	0.0854	0.0581	0.0126	0.0132	0.4729	0.3334	0.1572	0.1195	0.2300
	0.0832	0.2825	0.0658			0.5728	0.5537	0.0553	
**Corrected p values**									
Beta - Visual Task					*Beta - Procedural Task*				
	0.0108	0.0091	0.0070			0.2602	0.2259	0.2602	
0.0091	0.0167	0.0062	0.0062	0.0247	0.2259	0.3443	0.2602	0.2602	0.2732
0.1203	0.0294	0.0062	0.0062	0.0062	0.2602	0.2602	0.2602	0.2259	0.2732
0.0869	0.0944	0.0813	0.0230	0.0230	0.5226	0.3890	0.2602	0.2602	0.3018
	0.0944	0.2825	0.0864			0.5728	0.5728	0.2602	
**Channel reference**									
	Fp1	Fpz	Fp2			Fp1	Fpz	Fp2	
F7	F3	Fz	F4	F8	F7	F3	Fz	F4	F8
T7	C3	Cz	C4	T8	T7	C3	Cz	C4	T8
P7	P3	Pz	P4	P8	P7	P3	Pz	P4	P8
	O1	Oz	O2			O1	Oz	O2	

Supplementary Table 11. Uncorrected and corrected P-values for linear regressions associating Beta Power vs Change of task performance for each electrode.

**Uncorrected p values**									
Beta - Visual Task					*Beta - Procedural Task*				
	0.0421	0.0409	0.0498			0.0334	0.0060	0.0179	
0.0019	0.0109	0.0285	0.0188	0.0149	0.0391	0.0229	0.0188	0.0138	0.0255
0.0233	0.0015	0.0048	0.0019	0.0085	0.0039	0.0252	0.0033	0.0309	0.0501
0.2791	0.0014	0.0055	0.0012	0.1930	0.0075	0.0191	0.0817	0.0278	0.3804
	0.7228	0.3388	0.0930			0.0110	0.0007	0.0021	
**Corrected p values**									
Beta - Visual Task					*Beta - Procedural Task*				
	0.0590	0.0590	0.0654			0.0412	0.0253	0.0364	
0.0080	0.0254	0.0461	0.0359	0.0312	0.0456	0.0382	0.0364	0.0362	0.0382
0.0407	0.0080	0.0166	0.0080	0.0222	0.0204	0.0382	0.0204	0.0406	0.0553
0.3084	0.0080	0.0166	0.0080	0.2251	0.0264	0.0364	0.0858	0.0390	0.3804
	0.7228	0.3557	0.1149			0.0329	0.0148	0.0204	
**Channel reference**									
	Fp1	Fpz	Fp2			Fp1	Fpz	Fp2	
F7	F3	Fz	F4	F8	F7	F3	Fz	F4	F8
T7	C3	Cz	C4	T8	T7	C3	Cz	C4	T8
P7	P3	Pz	P4	P8	P7	P3	Pz	P4	P8
	O1	Oz	O2			O1	Oz	O2	
