# Supplementary material for: Auditory stimulation during REM sleep modulates REM electrophysiology and cognitive performance
Source: Commun Biol. 2024 Feb 16;7:193. doi: 10.1038/s42003-024-05825-2 (PMC10873307; doi:10.1038/s42003-024-05825-2)
Supplement: Supplementary file 3 — Description of Additional Supplementary Files [file 42003_2024_5825_MOESM3_ESM.pdf]

Description of Additional Supplementary Files

File name: Supplementary Data 1
Description: Numerical source data for Figures 1c-g, 2d, and 2f-g.
