# Supplementary material for: Auditory stimulation during REM sleep modulates REM electrophysiology and cognitive performance
Source: Commun Biol. 2024 Feb 16;7:193. doi: 10.1038/s42003-024-05825-2 (PMC10873307; doi:10.1038/s42003-024-05825-2)
Supplement: Supplementary file 5 — Reporting Summary [file 42003_2024_5825_MOESM5_ESM.pdf]

nature portfolio

Corresponding author(s): Penelope Lewis

Last updated by author(s): Jan 13, 2024

Reporting Summary

Nature Portfolio wishes to improve the reproducibility of the work that we publish. This form provides structure for consistency and transparency in reporting. For further information on Nature Portfolio policies, see our Editorial Policies and the Editorial Policy Checklist.

Statistics

For all statistical analyses, confirm that the following items are present in the figure legend, table legend, main text, or Methods section.

n/a	Confirmed	
☐	☒	The exact sample size (*n*) for each experimental group/condition, given as a discrete number and unit of measurement
☐	☒	A statement on whether measurements were taken from distinct samples or whether the same sample was measured repeatedly
☐	☒	The statistical test(s) used AND whether they are one- or two-sided<br>*Only common tests should be described solely by name; describe more complex techniques in the Methods section.*
☐	☒	A description of all covariates tested
☐	☒	A description of any assumptions or corrections, such as tests of normality and adjustment for multiple comparisons
☐	☒	A full description of the statistical parameters including central tendency (e.g. means) or other basic estimates (e.g. regression coefficient) AND variation (e.g. standard deviation) or associated estimates of uncertainty (e.g. confidence intervals)
☐	☒	For null hypothesis testing, the test statistic (e.g. *F*, *t*, *r*) with confidence intervals, effect sizes, degrees of freedom and *P* value noted<br>*Give P values as exact values whenever suitable.*
☒	☐	For Bayesian analysis, information on the choice of priors and Markov chain Monte Carlo settings
☐	☒	For hierarchical and complex designs, identification of the appropriate level for tests and full reporting of outcomes
☐	☒	Estimates of effect sizes (e.g. Cohen's *d*, Pearson's *r*), indicating how they were calculated

Our web collection on statistics for biologists contains articles on many of the points above.

Software and code

Policy information about availability of computer code

Data collection	Task and scripts were programmed in matlab.
Data analysis	Analysis scripts were custom programmed in Matlab. some EEG analysis was performed using FieldTrip (in Matlab)

For manuscripts utilizing custom algorithms or software that are central to the research but not yet described in published literature, software must be made available to editors and reviewers. We strongly encourage code deposition in a community repository (e.g. GitHub). See the Nature Portfolio guidelines for submitting code & software for further information.

Data

Policy information about availability of data

All manuscripts must include a data availability statement. This statement should provide the following information, where applicable:

- Accession codes, unique identifiers, or web links for publicly available datasets
- A description of any restrictions on data availability
- For clinical datasets or third party data, please ensure that the statement adheres to our policy

The data that support the findings of this study are available from OSF repository: https://osf.io/bjeq2/. Data for figure plots was included as Supplementary Data

Research involving human participants, their data, or biological material

Policy information about studies with human participants or human data. See also policy information about sex, gender (identity/presentation), and sexual orientation and race, ethnicity and racism.

Reporting on sex and gender	We recruited without regard to gender, and have reported the number in each gender.
Reporting on race, ethnicity, or other socially relevant groupings	We did not collect this information
Population characteristics	We have described age. Our participants were healthy and young, we screened to ensure no diseases that could influence sleep, and have not collected data on other diseases or conditions.
Recruitment	Participants were recruited through online and paper advertisements. It is likely that members of the University (whether staff or students) were more likely to volunteer due to their proximity.
Ethics oversight	Cardiff University School of Psychology Ethics Committee

Note that full information on the approval of the study protocol must also be provided in the manuscript.

Field-specific reporting

Please select the one below that is the best fit for your research. If you are not sure, read the appropriate sections before making your selection.

☐ Life sciences ☒ Behavioural & social sciences ☐ Ecological, evolutionary & environmental sciences

For a reference copy of the document with all sections, see nature.com/documents/nr-reporting-summary-flat.pdf

Behavioural & social sciences study design

All studies must disclose on these points even when the disclosure is negative.

Study description	Quantitave data collected in a within participant counterbalanced blinded crossover design.
Research sample	Healthy young people (18-35yrs) living within an easy commute of Cardiff University
Sampling strategy	Convenience sample
Data collection	Behavioural tasks were programmed in matlab and collected on a PC. EEG data was collected using a Brain Products system in the CUBRIC sleep lab.
Timing	Data collection started in January 2019 and ran until Feb 2020.
Data exclusions	Due to technical reasons, two participants did not complete the VDT testing in one of the four sessions. Therefore, these two subjects were excluded from the VDT analysis, but were included in the electrophysiological and behavioural analyses of the other tasks. Similarly, the polysomnographic recordings of one participant were not completed during the CNT night because of the midnight failure of the EEG system.
Non-participation	NA
Randomization	This was a within participant design.

Reporting for specific materials, systems and methods

We require information from authors about some types of materials, experimental systems and methods used in many studies. Here, indicate whether each material, system or method listed is relevant to your study. If you are not sure if a list item applies to your research, read the appropriate section before selecting a response.

Materials & experimental systems

n/a	Involved in the study
☒	☐ Antibodies
☒	☐ Eukaryotic cell lines
☒	☐ Palaeontology and archaeology
☒	☐ Animals and other organisms
☒	☐ Clinical data
☒	☐ Dual use research of concern
☒	☐ Plants

Methods

n/a	Involved in the study
☒	☐ ChIP-seq
☒	☐ Flow cytometry
☒	☐ MRI-based neuroimaging

Plants

Seed stocks	na
Novel plant genotypes	na
Authentication	na
